# Strain-based measurement system on an internal fixator for healing analysis of spinal defects through load redistribution

**DOI:** 10.3389/fbioe.2025.1543104

**Published:** 2025-03-31

**Authors:** Philip Johannes Steinbild, Nils Wieja, Luis Rodríguez Pino, Robert Gottwald, Anja Winkler, Stefan Zwingenberger, Alexander Carl Disch, Werner Schmölz, Niels Modler

**Affiliations:** ^1^ Institute of Lightweight Engineering and Polymer Technology, Faculty of Mechanical Science and Engineering, Dresden University of Technology, Dresden, Germany; ^2^ University Comprehensive Spine Center, University Center for Orthopaedics, Trauma and Plastic Surgery, University Hospital Carl Gustav Carus, Dresden, Germany; ^3^ Biomechanics Laboratory, Department of Orthopaedics and Traumatology, Medical University of Innsbruck, Innsbruck, Austria

**Keywords:** thoracolumbar spine, spinal fixation, strain measurement, bluetooth data transmission, healing simulation, internal fixator

## Abstract

A compact measurement system applied to an internal fixator for spinal bony healing is described and its usability for monitoring a simulated healing process on spine models is assessed. Four strain gauges were applied to carbon fiber reinforced polymer (CFRP) rods forming sensor integrated rods in order to measure a multitude of different strains using a minimum of strain gauges. This configuration enables the application not only on CFRP but also on rods made of a different material. Spinal didactic models were instrumented with the sensor rods in combination with pedicle screws in order to depict a bisegmental thoracolumbar stabilisation ranging from spine segments T11 to L1. The transmission of the acquired strain data to an evaluation computer was realized by a customized measurement system using an integrated Bluetooth chip. The measurement system is able to measure isolated bending and tensile or compression strain simultaneously. A standardized fenestration defect was induced at the middle section (T12). To mimic the healing process, three silicone discs of different Shore hardness (0 ShA, 8 ShA, 30 ShA) were inserted into the defect with 0 ShA simulating a fractured T12. A series of three spine models were tested under comparable conditions. Flexion bending moments of 5 Nm were applied to the specimens using a universal testing machine. Isolated bending strain on the rods increased from an unfractured state to the fractured state by 10%–27%. The healed state simulated by a silicone disc of 30 ShA lowered the isolated bending strain reliably for each specimen. Silicone discs of 8 ShA cannot simulate an intermediate healing state reliably.

## 1 Introduction

The treatment of spinal defects has been a longstanding challenge in the field of spinal surgery, necessitating innovative solutions to enhance treatment results and minimize complications. One way to treat spinal defects that are not sufficiently stable is to stabilize the spine with an internal fixator. Once the spinal defect has healed, the internal fixator should be removed.

Traditional methods of healing analysis have primarily relied on clinical assessments, radiographic imaging, and subjective patient-reported outcomes. While these approaches provide valuable information, they often fail to capture the dynamic biomechanical changes that occur during the healing process, resulting in high stress and radiation exposure to the patient and high treatment costs. In addition, due to artifacts or poor image quality, the data obtained from an X-ray image or a computed tomography (CT) scan may not be sufficient for the physician to make a decision. As medical professionals continue to refine treatment methods, there is a growing need for advanced measurement systems that can provide real-time insights into the biomechanical dynamics of the implant-treated spine. Such systems would enhance the quality of the treatment as well as the patient’s wellbeing. In this context, strain measurement systems have proven to be a promising approach because they provide a direct and quantitative way to evaluate the mechanical forces acting on implants (Waugh, 1966; Lightsey et al., 2021; Kim et al., 2022; Wang et al., 2024). Rohlmann et al. described a first device for *in vivo* measurements of loads on an internal fixator in 1994 Rohlmann et al. (1994). This device is based on an internal fixator first published by Dick in 1987 that was modified using an additional cylinder that featured 6 internal strain gauges (SGs) Dick (1987). Through calibration, this device was able to detect forces and moments that the internal fixator was subjected to. In the following years this device was used to measure forces and moments under different loading conditions such as during walking Rohlmann et al. (1997), sitting Rohlmann et al. (2001), and physical therapy Rohlmann et al. (2002). The results from these investigations are the base for the design data used in the development of modern internal fixator systems of various manufacturers. Although the device by Rohlmann et al. proved reliable and acquired high quality data, it is based on a design of an internal fixator from 1987, featuring a non-radiotransparent metal as a construction material and exhibiting very large geometrical dimensions compared to the internal fixator itself.

Apart from the measurement of forces and moments acting on an internal fixator, some work was done to monitor the fusion process of the spine during healing. In the publication by Szivek et al. (2005), calcium phosphate ceramic-(CPC)-coated SGs were applied to the right lamina of T9, T10, and T11, respectively. An additional non-coated SG was applied to the left rod of the internal fixator. The strain measurements were stopped after 37 days after surgery, however, a reduction of strains during the first 37 days of healing was recorded. On a self-developed spine model, Barri et al. described an approach of using a piezoelectric transducer in combination with a self-powered Fowler-Nordheim sensor data logger to monitor simulated fusion of the spine model (Barri et al. (2021), Barri et al. (2022)). Fusion was simulated using four different materials with varying Young’s modulus resembling different fusion stages. All three publications, however, lack the potential to identify and assess a certain load state in real-time. Additionally, the piezoelectric transducer used in Barri et al. (2021) and Barri et al. (2022) has a tendency to drift under static loading, making it only usable for dynamic load cases, or, like in this case, long term measurement. In Szivek et al. (2005) only one SG was placed on the rod, giving information about the strain at only one spot.

To be able to acquire real-time data of the biomechanical dynamics during the healing process and identify and assess load cases to give a feedback to the patient and medical professional, a measurement system needs to be developed that is compatible with many different internal fixator systems available on the market today. Additionally, it should feature materials that are radiotransparent. The article describes the feasibility study of a measuring system that is able to measure a multitude of different strains on internal fixator’s rods while using a minimum of sensors, leading to a small system that is applicable to almost any internal fixator configuration.

## 2 Materials and methods

### 2.1 Concept and load cases

**FIGURE 1:**
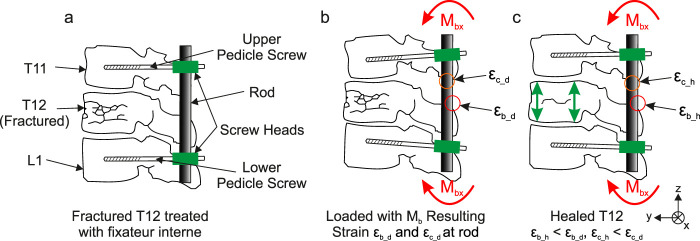
Schematic representation of sections T11 to L1 of a spinal column **(a)** treated with an internal fixator exhibiting a fracture in T12; loaded by a bending moment $M_{bx}$ (flexion): **(b)** during, and **(c)** after healing of fractured T12.

Limiting the complexity of the task, only a selected part of the spinal column is considered. The measurement system was developed for segments T11 to L1 with a bone defect to be analyzed in T12. It is assumed that during the healing process of T12, the load-bearing capacity of the fractured section will increase, causing a load redistribution from the internal fixator to T12. In consequence, this load redistribution causes a change in mechanical stress along the internal fixator’s rods, resulting in lower mechanical strains on the rod’s surface under load. To illustrate this behavior, Figure 1a shows a schematic representation of T11 to L1 with a fractured T12 treated with an internal fixator. When the structure is subjected to a bending moment $M_{bx}$ (Figure 1b) most of the load is carried by the rods. In addition to bending moments as illustrated in Figures 1b, c, compressive forces might act on the rods due to e.g., friction effects or body mass of the patient. The bending moments will cause bending strain $\varepsilon_{b\text{\_}d}$ on the outer edge of a rod while the compressive forces cause compressive strain $\varepsilon_{c\text{\_}d}$ , superimposing the bending strain (Figure 1b). After T12 has healed (Figure 1c), the same loading will cause a lower bending strain $\varepsilon_{b\text{\_}h}$ due to T12 carrying more load, distributing the moment $M_{bx}$ over a larger cross section. The same is to be expected with the compression strain, reducing from $\varepsilon_{c\text{\_}d}$ to $\varepsilon_{c\text{\_}h}$ . Measuring the change in mechanical strain of the rods during the time of healing can thus enable a quantitative evaluation of the damaged section. Because the strains shown in Figures 1b, c are constant along the z-axis between the two pedicle screws of each rod, they can be measured at almost any point on the rod surface. The position selected for this study is described below.

To be able to describe the deformation of the internal fixator rods, both need to be equipped with strain sensors. For the selection of strain sensors and their arrangement on the rods, the loading conditions need to be defined. With respect to the coordinate system of Figure 1, the load case “Flexion” loading the specimen with a bending moment of $M_{bx}$ was defined to be the basis for this study. The value of the moment is discussed in Section 2.3.

Due to the complex geometry and material properties of spine columns and resulting from the positioning of the installed implant, the effect of the loads on the internal fixator’s rods are a superimposed combination of strains resulting from bending, tension, and compression loading. Thus, three effects per rod need to be considered:

1. Strains resulting from bending moments with respect to the x-axis
2. Strains resulting from bending moments with respect to the y-axis
3. Strains resulting from pure tensile or compression loading.

In order to measure directional strain, strain gauges were selected to be the sensor of choice for the measurement system. Since relatively large bending strains of 2,000 ppm–3,000 ppm are expected, metallic SGs were chosen as they feature a higher measuring range and are substantially cheaper than semiconductor SGs.

The amount of SGs and their geometric arrangement on the rods as well as their electrical connection layout determine the kind of strain that can be measured using a Wheatstone bridge circuit. For measuring pure bending or tensile strains, a Wheatstone bridge circuit with two active SGs is chosen as a useful configuration for the application. For measuring bending strains, a half bridge layout is used while for the measurement of tensile or compression strains the diagonal bridge layout is used Hoffmann (2009). Thus, to measure the aforementioned three strain types, two SGs per Wheatstone bridge are required. In a classic measurement system, this configuration would result in six SGs per rod and twelve SGs per measurement system.

**FIGURE 2:**
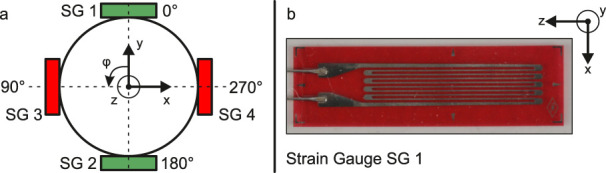
Schematic representation of the cross-section of an internal fixator rod with positioning of the strain gauges (SG) **(a)** and SG 1 with coordinate system for defining the orientation **(b)**.

There are some restrictions to the design of the system. The commercial internal fixator system used for this study features a rod diameter of 6 mm. This diameter is determining the available circumference and surface area for the positioning of the SGs. The width of the backing of commercially available SGs can be chosen as low as 2.5 mm. Each SG is oriented with the measuring grid pointed in z-direction for best sensitivity, compare Figure 2b. The SGs are placed in the middle of each rod, half way between the pedicle screws, in order to measure strains in an undisturbed area. The half bridge layout requires that the SGs are positioned opposite of each other on the surface of the rod. This results in that the four SGs for measuring bending strain need to be positioned one at each quarter of the rod at 0°, 90°, 180° and 270° of the circumference. With a width of the backing of 2.5 mm per SG, there is only approximately 2.2 mm left for each additional SG. Thus the SGs for measuring tensile and compression strains need to be positioned above or below the bending SGs. To solve this problem, the proposed electronic device should be able to switch from a half bridge to a diagonal layout, so that the four SGs can be used for two different measurement modes:

• Mode 1: Two half bridge layout circuits, measuring strains resulting from isolated bending relative to the x- and y-axis

• Mode 2: Two diagonal bridge layout circuits, measuring strains resulting from isolated tensile or compression loading. The two measurands are then averaged.

Due to the interconnection in a half bridge layout for measuring isolated bending strains (mode 1) the impact of temperature changes on the measurement is inherently compensated Hoffmann (2009). For a diagonal bridge layout, temperature changes can only be compensated if additional SGs are integrated into the Wheatstone bridge circuit that are not loaded mechanically. This is not the case in the system used here. However, in this study, the measurements are taken in a temperature controlled laboratory at a stable 23°C. Thus, the measurements taken for this study were not effected by temperature changes. The resulting SG layout on the circumference of each rod is shown schematically in Figure 2a.

### 2.2 Materials

The anatomical model Spine Lumbar T10-L5 Sacrum LSS9370 (Synbone AG, Zizers, Switzerland) was selected as a reference model for the development of the measurement system. This model is supposed to have sufficient anatomical correctness and mechanical properties to withstand the forces it will be subjected to during the development process. For the following tests, the model was reduced to a segment from T11 to L1 by cutting away the sections T10 and L2 to sacrum.

Modern internal fixator systems from commercial manufacturers like Stryker Corporation (Kalamazoo, MI, USA) and Coligne AG (Zurich, Switzerland) consist of pedicle screws made of titanium and connecting rods made of titanium or carbon fiber reinforced polymer (CFRP). For the present study, the internal fixator system flexStaas 3 (Coligne AG) was selected. This enables the assessment of the compatibility of the developed measurement system with rods made of CFRP, which is considered to be the state of the art material for medical applications. In this case the proprietary material ostaPek (Coligne AG) is used.

**FIGURE 3:**
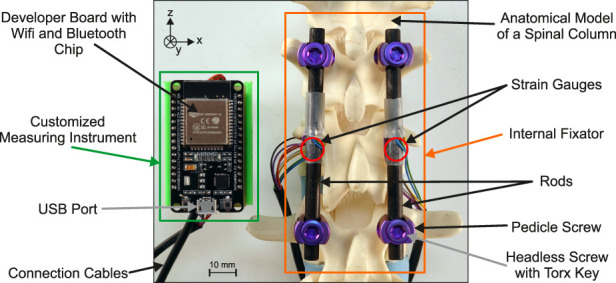
Complete measurement system comprised of the customized measuring instrument and the internal fixator with adhesively bonded strain gauges installed on the anatomical model.

The base configuration of the selected system flexStaas 3 consists of four pedicle screws, two rods, and four headless screws with torx key to fasten the rods in the multiaxial head of the pedicle screw. The complete measurement system applied to the segment from T11 to L1 of the anatomical model is shown in Figure 3. The internal fixator system is installed on the anatomical model as intended by the manufacturer. The SGs are situated on the surface of the rods halfway between the pedicle screws. For the SGs, Goblet FLGB-1 (Tokyo Measuring Instruments Laboratory Co., Ltd., Tokyo, Japan) were chosen since they fulfill the geometry requirements defined in section 2.1 (backing width 2.5 mm and backing length 6 mm, compare Figure 2b). Two cables connect the SGs to the measurement system.

**FIGURE 4:**
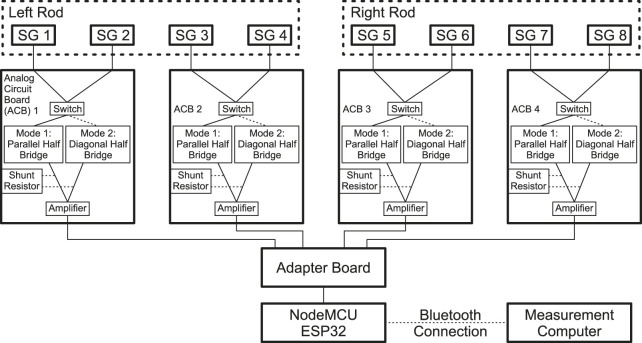
Schematic circuit diagram of the customized measuring instrument.

The strains resulting from the loading of the rods are measured with a customized measuring instrument. The electronic circuit is shown in the schematic diagram in Figure 4. The instrument consists of six printed circuit boards (PCB): 1 ESP32 (NodeMCU ESP32, SIMAC Electronics GmbH, Neukirchen-Vluyn, Germany), 1 adapter board, and 4 analog circuit boards (ACB). The ACBs are responsible for acquiring the electric signals coming from the SGs and amplify them. Using a switch (FSA2267, Fairchild Semiconductor Corporation, Sunnyvale, CA, USA), it is possible to quickly change from Mode 1 to Mode 2 measurements. The signal is amplified using an instrumentation amplifier (INA332, Texas Instruments Incorporated, Dallas, TX, USA). The configuration using 4 individual ACBs was chosen so that faulty PCBs could easily be replaced. The analog signals are digitized and transferred to the development board via the adapter board, which was custom designed. Using the Bluetooth chip on the ESP32, the data is then sent to a computer. Electric energy is supplied using the USB port on the development board, however, also other forms of energy supply are possible. The system achieves an acquisition rate of 4 Hz, including the time needed to switch from mode 1 to 2 and back. Each time the system starts up, the measurement is zeroed and a shunt calibration is performed to balance the Wheatstone bridge and eliminate any systematic errors. If necessary, zeroing and shunt calibration can be performed during operation.

The calculated effective noise level of the amplified signal is 290 µVrms. This noise level is negligible when considered in the context of the analog-to-digital converter of the ESP32, which is configured to have a quantization step of 806 µVrms. Given the integral nonlinearity of the ESP32 of 12 LSB, the measurement error would be between 0.3% and 0.8% for the voltage measurement. However, given that the system is initialized to zero at startup and a measurement is invariably a differential value between the initialized value and the actual measurement, this error is compensated for. The relevant error is the quantization error of 0.5 LSB, which, in the context of strain measurement, is equivalent to 3.7 ppm. Therefore, all absolute strain values given in this article are rounded to the nearest ten.

Both measurement modes 1 and 2 are compensated so that isolated bending (mode 1) and isolated tension/compression strain (mode 2) can be measured independently. However, a systematic error is introduced into the measurement due to slight imperfections in the placement and orientation of the SGs on the rod. This systematic measurement error is relatively small, but for small measurement values it means that they are no longer meaningful. If a rod experiences large bending strains while at the same time experiencing relatively small tensile strains, the systematic error will have a large effect on the reading of the small tensile strain reading.

### 2.3 Methods

The developed measurement system is used to experimentally investigate whether it is possible to recognize different stages of the healing process of an implant-treated spinal segment model. Therefore, a method simulating a fracture and the subsequent healing process within T12 similar to the simulation method in Barri et al. (2021) was used. Immediately after a vertebral fracture occurs, its rigidity is reduced. During healing, the tissue regains structural integrity, increasing its rigidity.

The anatomical model (Spine Lumbar T10-L5 Sacrum LSS9370, Synbone AG) used in this study consists of three different materials. The vertebral bodies are made of a high-density polymer foam, resembling the average density of human bone. The intervertebral discs are made of a low-density polymer foam. The construction is held together by a belt made from imitation leather. This belt was completely removed during specimen preparation, leaving only vertebral bodies and intervertebral discs bonded with adhesive. This results in a very flexible construct that resembles the geometry and, to some extent, also the mechanical behavior of a human spine that is stripped of any tissue surrounding the spine, e.g., ligaments or muscles.

**FIGURE 5:**
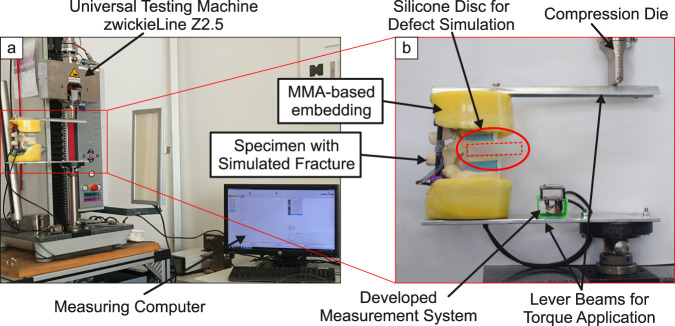
Complete measurement setup for loading the specimens **(a)** and lever beams for application of bending torque with clamped specimen **(b)**.

To prepare a specimen, the anatomical model is reduced to vertebral bodies T11 to L1 and intervertebral discs. The resulting construct is then equipped with the instrumented internal fixator like in Figure 3. In the following, this state is referred to as the intact state. In order to simulate a fracture, part of the corpus of the anatomical model’s T12 was then cut out over a height of 10 mm, leaving enough material at the posterior side, so that the vertebral body does not collapse. This reduction in the cross section of the anatomical model’s T12 results in a substantial decrease in the rigidity of the component, while only a minor reduction is observed in the rigidity of the entire construct. Thus, only a small change in rigidity needs to be regained by the method used to simulate the healing of T12. Nevertheless, the rigidity of the polymer vertebral body needs to be increased gradually in order to simulate the course of healing of the vertebra. The simulation of the course of healing is done by using three discs made of silicone (Polymerschmiede GmbH, Moenchengladbach, Germany), one for each stage of healing. The three discs are made from silicone of different Shore hardness 0 ShA, 8 ShA and 30 ShA, respectively. The different mechanical properties of the three discs allow a slight increase in the stiffness of the structure, which is necessary to show the sensitivity of the measurement system to slight increases in the stiffness of a spinal component, even if it is only an anatomical model. The position of the silicone in the T12 is shown in Figure 5b, highlighted by the dotted red line.

To load the specimen, a universal test machine zwickieLine Z2.5 (ZwickRoell GmbH & Co. KG, Ulm, Germany) was used (Figure 5a). Simple lever beams were used to apply a bending moment to the specimen. Sections T11 and L1 were embedded in a methyl methacrylate-(MMA)-based polymer (Technovit^®^3040, Kulzer GmbH, Hanau, Germany, yellow color). The levers were bolted to the polymer embedding (Figure 5b). During loading, the compression die exerts a downward force on the top of the lever beam, inducing not only a moment but also eccentrically acting compressive forces on the specimen. The effect of these resulting lateral forces on the measurands are seen as small compared to the moment.

In Rohlmann et al. (2009), flexion was simulated by applying loads to the L1 vertebra. The relevant load case for the simulation in the study at hand would be (B), an eccentric force loading the specimen using a pure moment of 7.5 Nm. However, the anatomical models used in this study are made from polymer foam and the absence of ligaments or muscles result in very flexible specimens. A bending moment of 7.5 Nm leads to very high deformations and strains, making the measurements of the different simulated healing states not comparable. Therefore, a total of three of the aforementioned anatomical spine models are tested for the load case flexion with a maximum bending moment of $M_{bx}=$ −5 Nm (compare coordinate system of Figure 3). The length of the lever is 185 mm. A compression force of 27 N was brought onto the lever mechanism for each measurement to induce the desired bending moment.

Each of the three spine model is tested in four states, following a simulated fracture and healing process:

1. Intact without simulated fracture,
2. Simulated fracture with silicone 0 ShA, resembling a new fracture,
3. Simulated fracture with silicone 8 ShA, as a first healing stage, and
4. Simulated fracture with silicone 30 ShA, as a second healing stage.

The cross-section of the spine tapers from the bottom to the top. For this reason, and also because the vertebral bodies have different individual geometries, slight deviations in the orientation of the rods within the pedicle screws are common. Therefore, the applied bending moment results in bending strain not only in x-direction, but also in the y-direction. Thus, the absolute value of the bending strain $\varepsilon_{b}$ was calculated using Equation 1 with $\varepsilon_{bx}$ being the bending strain measured using SG 1 and SG 2 and $\varepsilon_{by}$ being the bending strain measured using SG 3 and SG 4 (Figure 2).

$$\varepsilon_{b}=\sqrt{\varepsilon_{bx}^{2}+\varepsilon_{by}^{2}}$$ (1)

As described in Section 2.1, mode 2 measurements result in two measurands per rod resembling strain resulting from pure tensile or compression loading. The pure tensile or compression strain per rod $\varepsilon_{c}$ is calculated using Equation 2 with $\varepsilon_{c1}$ measured using SG 1 and SG 2 and $\varepsilon_{c2}$ measured using SG 3 and SG 4.

$$\varepsilon_{c}=\frac{\varepsilon_{c1}+\varepsilon_{c2}}{2}$$ (2)

## 3 Results

The resulting absolute values of the bending strain $\varepsilon_{b}$ and compression strain $\varepsilon_{c}$ for all three spine models under a load of $M_{bx}=$ −5 Nm are shown in Table 1 for the intact state. The mean values of bending and compression strain are also shown in Table 1.

**TABLE 1:** Maximum bending $\left(\varepsilon_{b,i}\right)$ and compression $\left(\varepsilon_{c,i}\right)$ strain on the rods of spine models i=1,2,3 during simulated flexion in intact state.

	$\varepsilon_{b,i}$ [ppm]/ $\varepsilon_{c,i}$ [ppm]			
Spine Model (i)	1	2	3	Mean values
Left Rod	2380/40	1890/10	1810/60	2027/37
Right Rod	2100/120	2170/90	2120/120	2130/110

The maximum compression strains of Table 1 are very small compared to their maximum bending counterparts (0.5%–5.7%). The systematic error described at the end of section 2.2 might have a big impact on these small strain measurements. Therefore, the measurements of compression strains are not considered further in this study. However, the small readings indicate that the loading using a simple lever (Figure 5) does not cause any significant lateral forces that result in high isolated tension or compression strains.

**FIGURE 6:**
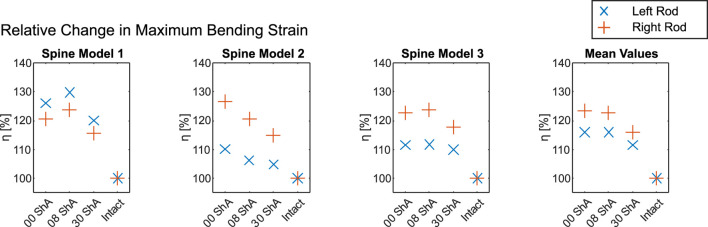
Change in maximum bending strain relative to the intact state (Table 1) over the course of a simulated fracture (0 ShA) and healing process (8 ShA and 30 ShA) for each spine model tested (1–3) and mean values.

In Figure 6 the change in the absolute maximum bending strain of the different simulated healing states relative to the intact state η is given. η is calculated using Equation 3.

$$\eta =\frac{\Delta \varepsilon_{b}}{\varepsilon_{b,i}}=\frac{\varepsilon_{b,f}-\varepsilon_{b,i}}{\varepsilon_{b,i}}=\frac{\varepsilon_{b,f}}{\varepsilon_{b,i}}-1$$ (3)

with $\varepsilon_{b,i}$ from Table 1 and $\varepsilon_{b,f}$ being the maximum strain of the respective simulated healing states for bending (b).

Figure 6 shows the change in maximum bending strain. For bending, all three spine models exhibit a significant increase in bending strain after the introduction of the defect. However, only spine model 2 shows the expected effect, where the simulated state of a new fracture (0 ShA) shows the highest strain for both rods (left rod 110.1%, right rod 126.6%), which decreases with simulated healing to 106.1% and 120.5% for 8 ShA and finally 104.7% and 114.9% for 30 ShA. For spine models 1 and 3 the highest strain on each rod was measured at the first healing stage of 8 ShA. Looking at the mean values in Figure 6, the simulated state of a new fracture (0 ShA) and the first healing stage (8 ShA) show almost the same changes in strain, while the second healing stage (30 ShA) shows significantly lower values.

## 4 Discussion

### 4.1 Measurement system

The unique characteristic of the measurement system described in Section 2.2 is the ability to measure bending and tension/compression strains separately by using two modes that can be switched within a short time. This leads to a reduced number of 4 SGs for a measuring task that normally needs at least 6 SGs. With information on the mechanical behavior of the rods, i.e., the Young’s modulus, it is possible to calculate bending moments $M_{bx}$ and $M_{by}$ as well as the axial force $F_{z}$ acting along the z-axis of each rod. The system described in Rohlmann et al. (1994) enables the detection, respectively the calculation, of all six load components $\left(F_{x},F_{y},F_{z},M_{x},M_{y},M_{z}\right)$ acting on each rod. However, a calibration needs to be done in order to determine all 36 calibration constants, complicating the preparation of the device greatly. Whether it is necessary to measure all six load components to assess the healing status of a spine remains an open question that needs to be answered in either studies using cadaveric specimens or clinical studies. The results shown in Section 3 indicate that measurement and comparison of bending strains might be sufficient, especially since patients are only capable of subjecting the internal fixator to a limited number of load cases.

For the measurement system described in this publication, classic metallic strain gauges were used. The relatively low sensitivity was compensated by using low-noise amplifiers, enabling a cost-effective approach for the system. In contrast, the system described in Rohlmann et al. (1994) uses semiconductor SGs, which do not require high amplification, due to high sensitivity of the sensors themselves, but are cost-intensive. This lowers the possibility for the system to be developed into a commercial system for healing assessment. In Szivek et al. (2005), the exact type of SG is not mentioned.

### 4.2 Form factor and data transmission

Although relatively large in geometry, the telemetry system of Rohlmann et al. (1994) needed to power the sensors and record and transmit data via a wireless connection. This functionality was integrated into the device. The customized measuring instrument (Figures 3, 4) features a small form factor and wireless data transmission. However, there is still great potential for further miniaturization and integration to enable implantation in animals or even humans. For this study, the system was powered using a battery. The miniaturization and integration step could also enable energy harvesting for a completely autonomous system or energy transfer via induction like in Rohlmann et al. (1994).

### 4.3 Acquisition rate

The relatively low acquisition rate of 4 Hz is primarily caused by switching from mode 1 to mode 2 and *vice versa*. Each time the measurement mode is changed, the system measures a peak that needs time to level out to the correct reading. Additionally, device-internal averaging of the measurands is used to minimize noise, which also slightly reduces the acquisition rate. Further optimization could also improve the time it takes for the signal to level off, resulting in higher acquisition rates. For the relatively slow movements expected during healing, however, the acquisition rate of 4 Hz seems to be sufficient.

### 4.4 Healing simulation and measured data

The presented data indicates a usability of the described measurement system for monitoring spinal fracture healing or even spinal fusion. All measured bending strains on the internal fixator’s rods increased after the introduction of a simulated fracture. Of the three spine models tested, only one, spine model 2, showed the expected behavior: simulating three different healing states using silicone discs of changing Shore hardness, a reduction of isolated bending strain from a silicone disc with 0 ShA over 8 ShA to 30 ShA was observed while loaded with a bending moment of 5 Nm. For spine models 1 and 3 the least healed state simulated using a silicone disc of 0 ShA showed lower strains than the simulation with 8 ShA. However, the simulation with 30 ShA showed substantially less strain than the simulation with 0 ShA and 8 ShA for both rods. The mean values in Figure 6 emphasize the tendency to allow no clear indication for 0 ShA and 8 ShA while 30 ShA causes significantly lower bending strain.

The differences in stiffness between the silicone discs with 0 ShA and 8 ShA appear to be too small to have a significant impact on the overall rigidity of the specimens, resulting in a higher or constant level of strain when going from 0 ShA to 8 ShA. In Qi et al. (2003) and Larson (2019), the difference in Young’s modulus for silicones with Shore hardness of 0 ShA and 8 ShA is very small, which supports this inference. Silicones with a Shore hardness of 30 ShA show a significantly higher Young’s modulus. For the setup in this study, this significantly higher Young’s modulus results in a higher rigidity of the specimens. This behavior might be superimposed by setting processes of the material combination of vertebral bodies made of high-density polymer foam and intervertebral discs made of low-density polymer foam partially connected by pedicle screws and CFRP rods.

The following conditions were identified to have a significant influence on the absolute values of the measurements:

• Position of the pedicle screws in the vertebra

• screw-in depth of the pedicle screws in the vertebra

• geometry of the cut in T12 and

• amount of preload applied during installation of the internal fixator system.

Even small differences in these conditions result in a significant change of the mechanical behavior of each specimen. In this study, this effect can be observed for the values of maximum bending strain of the left rod in Table 1. This left rod of spine model 1 shows a significantly higher maximum bending strain than spine models 2 and 3. However, in a real-world application on a patient, a perfectly symmetrical assembly of the internal fixator is compromised by the individual geometry of the spine and the above mentioned installation conditions. Furthermore, load redistribution during the early stages of healing may result in a weakening of the spine before the rigidity of the tissue increases, leading to increased strain. This confirms that the recorded data should always be viewed as relative changes compared to the fractured (or intact) state, rather than as absolute values.The measurement of isolated compression strain is subject to systematic errors when the measured values are as small as in this study. In order to obtain reliable readings where superimposing strains do not have an impact on small strains, an additional error analysis or a calibration is necessary. This was not the focus of this study.

## 5 Conclusion

Within this article, a new integrable measurement system that is able to measure strains on rods of an internal fixator was described and tested. The system consists of four metallic strain gauges per rod that are used in two modes to simultaneously measure strains resulting from isolated bending and isolated tensile or compression loading. It shows high sensitivity and sufficient acquisition rate while featuring a small form factor and only 4 SGs per rod. The measurement system was tested on three spine models with simulated healing after a fracture.

The three different stages of healing were simulated using silicone discs with three different Shore hardness. The results showed that a very small increase in rigidity of the specimen using a silicone disc of 8 ShA cannot be identified reliably judging from the measurements of isolated bending strain. However, a slightly higher increase in rigidity using a silicone disc of 30 ShA showed significantly lower strain on the internal fixator’s rods across all specimens. This behavior serves as an indication of the efficacy of the measurement system and the method of simulating the course of healing. It is noteworthy that the utilized anatomical spine model is devoid of ligaments and muscles, thereby exhibiting a considerably bigger compliance. Nevertheless, a reliable simulation of the healing process through the implementation of relatively straightforward methodologies is possible using this study’s approach, particularly when compared to the complex procedures employed on specimens of human tissue. This enables development of new methods for healing assessment to be done in laboratory environments with polymer models rather than expensive human cadaver specimens.

The measurement system has been tested for a segment from T11 to L1. However, when modified appropriately, it can be used in any segment of the human spine when screw-rod systems are used in cases of spinal instability. This is made possible by using only 4 small metallic SGs that can be applied to any rod made from CFRP, titanium, or even stainless steel, while also being able to simultaneously measure isolated bending strain and isolated tensile or compression strain. Due to the modular approach of the measurement system, the necessary PCBs can be placed independently, allowing an adaptation to almost any installation space available. Comparing the presented approach to measurement systems in literature described in section 1 and discussed in section 4.1, the measurement system shows advantages in regard to simplicity, adaptability, and cost-effectiveness while reducing the need for calibration and the amount of data.

This article describes a feasibility study of a measurement system capable of measuring a variety of different strains on internal fixator rods while using a minimum of sensors, resulting in a small system that is applicable to almost any internal fixator configuration. With further development and miniaturization the system has the potential to be implantable in humans. The system can be zeroed and shunt-calibrated, balancing the Wheatstone bridge anytime in order to allow relative measurements to a defined state. In application, this defined state could be a posture of the patient that marks the beginning of a physical test and measurement.

In addition, the article demonstrates a method to mechanically mimic the healing process of a vertebral body of a human spine using cheap and readily available materials, allowing the avoidance of cadaveric studies and the waste of valuable cadaveric specimens in the early stages of measurement system development. Further research is needed, however, to clarify and define the boundary conditions and loads in more detail.

## Data Availability

Links to the datasheets of materials used in this article can be found in a PDF-file in the supplementary material.
