# Supplementary material for: Strain-based measurement system on an internal fixator for healing analysis of spinal defects through load redistribution
Source: Front Bioeng Biotechnol. 2025 Mar 31;13:1543104. doi: 10.3389/fbioe.2025.1543104 (PMC11994651; doi:10.3389/fbioe.2025.1543104)
Supplement: Supplementary file 1 [file DataSheet1.pdf]

Links to Data Sheets

Technovit 3040, Kulzer GmbH, Hanau, Germany: https://kulzer-technik.com/media/product-downloads/kulzer-technik/produktinformation/catalog-materialography-12-2022.pdf

flexStaas 3, Coligne AG, Zurich, Switzerland: data sheet available upon request: https://spinenuances.com/contact-spinenuances/

NodeMCU ESP32, SIMAC Electronics GmbH, Neukirchen-Vluyn, Germany: https://joy-it.net/files/files/Produkte/SBC-NodeMCU-ESP32/SBC-NodeMCU-ESP32_Datasheet_2023-09-13.pdf

FSA2267, Fairchild Semiconductor Corporation, Sunnyvale, CA, USA: https://www.mouser.com/datasheet/2/149/FSA2267A-188925.pdf?srsltid=AfmBOooxlIZLLmNEoJ_O8vlwLc9SqIM6zlmmX72SaA6U6M_ilY3TAhSX

Goblet FLGB-1, Tokyo Measuring Instruments Laboratory Co., Ltd., Tokyo, Japan: https://tml.jp/eng/documents/strain_gauge/Fseries.pdf

INA332, Texas Instruments Incorporated, Dallas, TX, USA: https://www.ti.com/lit/ds/symlink/ina332.pdf?ts=1742754467059
